# Spectrum of neuropathic skeletal injuries in children: a pictorial essay

**DOI:** 10.1186/s13244-025-01920-y

**Published:** 2025-03-07

**Authors:** Maria Chiara Bonanno, Aurélie O’Keane, Pierre Mary, Anca Tanase, Marianne Alison, Eléonore Blondiaux, Hubert Ducou le Pointe, François Chalard

**Affiliations:** 1 Department of Pediatric Radiology, Armand Trousseau Hospital, AP-HP, Paris, France; 2 Department of Pediatric Orthopedic and Reconstructive Surgery, Armand Trousseau Hospital, AP-HP, Paris, France; 3 Department of Pediatric Radiology, Robert-Debré Hospital, AP-HP, Paris, France

**Keywords:** Neuropathic arthropathy, Charcot, Congenital insensitivity to pain, Spinal dysraphisms

## Abstract

**Abstract:**

Neuropathic skeletal injuries in children are common manifestations of conditions associated with sensory impairment. The underlying aetiologies may be rare entities such as congenital insensitivity to pain or more prevalent disorders such as spinal dysraphisms. While the imaging manifestations of such injuries have been described in adults, the paediatric literature is sparse, primarily comprising case reports and case series with insufficient imaging data. Characteristic imaging findings in patients with neuropathic skeletal injuries include neuropathic arthropathy, avascular necrosis, joint dislocation, repeated fractures with exuberant callus formation, epiphyseal separation, and acro-osteolysis. Conventional radiography, MRI, and CT all contribute to the comprehensive assessment of paediatric neuropathic lesions. This pictorial essay illustrates the spectrum of imaging findings in children with neuropathic skeletal injuries of varying aetiologies as well as their natural evolution and treatment.

**Critical relevance statement:**

This essay addresses a critical gap in the literature on paediatric neuropathic skeletal injuries, providing a detailed overview of their imaging manifestations, natural progression, and relevant treatment strategies, through contemporary imaging techniques such as radiography, MRI, and CT.

**Key Points:**

- Unrecognised neuropathic skeletal injuries cause progressive permanent deformities, impacting quality of life.
- Suspect neuropathic arthropathy in children with sensory loss presenting with painless inflamed joints.
- Neuropathic injuries may be mistaken for osteomyelitis, septic arthritis, or other disorders.

**Graphical Abstract:**

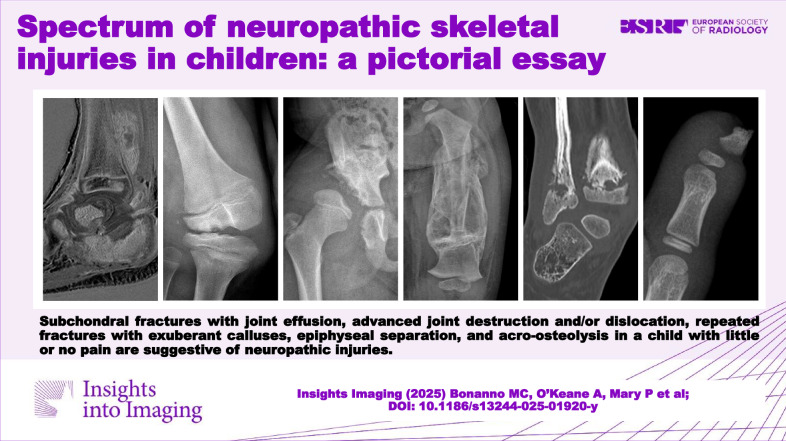

## Introduction

Neuropathic skeletal injuries in children are secondary to disorders that affect the peripheral nervous system causing sensory loss, such as congenital insensitivity to pain (CIP) and spinal dysraphisms (SDs) [1]. Although these conditions are rare, the resulting musculoskeletal injuries are a common orthopaedic manifestation in the affected population. A child with CIP will experience multiple damages to different joints and skeletal segments throughout life [2]. These injuries typically occur after minor trauma and go unrecognised because of the sensory deficit and the abnormal response to pain. Untreated, they can lead to severe disability, so imaging plays an important role in diagnosis.

To our knowledge, data are lacking on the prevalence of skeletal neuropathic injuries in children. By querying the database of our institutions, Hôpital Armand-Trousseau and Hôpital Robert-Debré (Paris) we found ten children who received treatment for this type of anomaly between 2010 and 2024. The clinical and imaging histories of the patients revealed multiple fractures, avascular necrosis (AVN), neuropathic arthropathy (NA), joint dislocation, epiphyseal separation, septic arthritis, and acro-osteolysis (Table 1). Six children had congenital insensitivity to pain, three children had surgery for SD at birth, and one child had lower-extremity sensory impairment following cerebral palsy.

**Table 1:** Clinical and imaging history of ten children who received treatment for skeletal neuropathic injuries between 2010 and 2024 in Hôpital Armand-Trousseau and Hôpital Robert-Debré (Paris)

Patient	Sex	Diagnosis	Location	Type of injury	Age at injury (years)	Treatment	Outcome
1	M	CIP with genetic diagnosis of HSAN IV (AR NTRK1 mutation)	Both hands	Acro-osteolysis of the distal phalanges	2–8	None	Healed
			L Tibia	Mid-shaft fracture	3	Cast and immobilisation	Healed
			L Ankle	NA	3	Orthosis	Healed
			L Knee	Lateral condyle AVN	5	Femoral varus osteotomy and hemi-epiphysiodesis	Walks with orthosis
			R Ankle	NA	6	Orthosis	Deformity, walks with orthopaedic shoes
			Spine	NA	15	Posterior T4-L4 fusion	Stable
2	M	CIP, familial diagnosis	R Shoulder	Epiphyseal separation and dislocation	5	Cast and immobilisation	Deformity
			R Hip	Joint dislocation	8	Watchful waiting	Stable
			R Knee	Lateral condyle AVN	9	Femoral varus osteotomy	Persistent deformity, walks with orthosis
			L Knee	Lateral condyle AVN	10	Orthosis	Healed
3	M	CIP, familial diagnosis	L Knee	Lateral condyle AVN	9	Total arthroplasty after failure of hemi-epiphysiodesis	Above-the-knee amputation
4	M	CIP with genetic diagnosis of HSAN IV (AR NTRK1 mutation)	L Knee	NA	3	Tibial valgus osteotomy	Leg-length discrepancy, waiting for surgery
			R Calcaneus	Fracture	5	Cast and immobilisation	Healed
			L Ankle	NA	7	Orthosis	Deformity
			R Ankle	Epiphyseal separation	14	Cast and immobilisation	Healed
5	M	CIP, no genetic mutation identified	R Hip	Recurrent septic arthritis and femoral neck fracture	5	Multiple arthrotomies and cast immobilisation	Walks with orthosis
6	M	CIP, clinical diagnosis	R Elbow	NA with recurrent septic arthritis	1	Multiple arthrotomies, synovectomy and cast immobilisation	Deformity
			L Femur	Mid-shaft fracture	1	Cast and immobilisation	Healed
			R Ankle	NA	3	Orthosis	Healed
			L Ankle	NA	9	Orthosis	Walks with orthosis
			L Elbow	NA	9	Watchful waiting	Healed
7	F	Meningocele (L LL sensory deficit)	L Ankle	NA	7	Walking boot and crutches	Ongoing follow-up
8	F	Lipomyelomeningocele (L LL sensory deficit)	L Hip	NA and dislocation	3	Femoral osteotomy and acetabuloplasty	Healed, tibial lengthening surgery
9	F	Myelomeningocele (R LL sensory deficit)	R Ankle	Epiphyseal separation	9	Splint immobilisation	Healed
10	M	Cerebral palsy	L Knee	Lateral condyle AVN	7	Watchful waiting	Healed

Patients 1 and 2 are siblings, with a genetic diagnosis confirmed only in the older brother (Patient 1). Similarly, Patients 3 and 4 are siblings, with a genetic diagnosis confirmed only in the younger brother (Patient 4)

*AR* autosomal recessive, *AVN* avascular necrosis, *CIP* congenital insensitivity to pain, *HSAN IV* hereditary sensory and autonomic neuropathy type IV, *L* left, *LL* lower limb, *NA* neuropathic arthropathy, *NTRK1* neurotrophic tyrosine kinase receptor type1, *R* right

This pictorial essay illustrates the spectrum of imaging findings in children with neuropathic skeletal injuries of varying aetiologies as well as their natural evolution and treatment.

## Causes of neuropathic injuries in children

Congenital insensitivity to pain (CIP) is an umbrella term referring to a phenotypic manifestation of a heterogeneous group of extremely rare autosomal dominant and recessive disorders that are collectively called hereditary sensory and autonomic neuropathy (HSAN). These disorders are classified according to genetic mutation, inheritance pattern, and clinical features in eight main types [3]. The prevalence of the CIP phenotype remains unknown, but it is estimated to be one case in one million based on known cases in the United Kingdom [4].

HSAN involves peripheral nerve fibres, specifically affecting small unmyelinated fibres more than large myelinated ones, resulting in a loss of multimodal sensation and autonomic dysfunction [3]. As a consequence, these patients experience various degrees of loss of pressure, proprioception, vibration, temperature and pain sensation, as well as symptoms of dysautonomia, such as sweating, and vasomotor and temperature disturbances, which results in recurrent episodic fever and hyperthermia [5].

Because of the frequency of musculoskeletal involvement, these disorders were first described as syndromes with bizarre skeletal deformities [6]. The loss of pain sensation in these patients results in complications of inadvertent repeated microtrauma, such as NA and AVN, and painless injury, such as fractures, infections and auto-amputation.

Although most published descriptions of orthopaedic manifestations of HSAN are fragmented and are mostly case reports [7–12], some authors have studied populations of 13 to 20 patients providing a more thorough list of lesions that align with the manifestations observed in our population [2, 13, 14].

Spinal dysraphisms (SDs) are a heterogeneous group of congenital malformations of the spinal cord with a wide range of clinical manifestations. For example, patients with myelomeningocele have morbidities related to lower-limb sensory and motor impairments, ranging from muscular weakness to complete paraplegia and from hypoesthesia to anaesthesia, as well as spinal and foot deformities [15]. SDs are considered the most common cause of NA in children, with an incidence of 1.05% to 1.42% [16–18].

Other causes of neuropathic injuries in children include peripheral nerve damage, cerebral palsy, neurofibromatosis, spinal neuroblastoma, and syringomyelia [16, 17, 19, 20]. These aetiologies remain extremely rare, with only a few case reports of children.

In adults and older patients, NA is commonly accepted as a specific pathology with secondary neuropathy caused by metabolic diseases, such as diabetes and chronic alcoholism; infections, such as tertiary syphilis and leprosy; and spinal cord pathology [21]. Recently, some iatrogenic causes of NA in adults have been added to the list, with reports of NA induced by trauma during spinal surgery and by monoclonal therapy [22, 23]. Nonetheless, adult aetiologies are not found in children.

## Imaging modalities

Conventional radiography, MRI, and CT all play pivotal roles in the workup, follow-up, and treatment planning of paediatric neuropathic injuries.

Radiographs are the first-line assessment due to their wide availability, rapid acquisition, cost-effectiveness, and sensitivity in visualising skeletal pathology. Radiographs are essential for instant diagnosis, especially because neuropathic injuries can be asymptomatic or have vague symptoms. They should be performed at any sign of concern, such as redness, swelling, or functional impairment, even without a clear trauma history.

MRI is crucial for deeper lesion analysis, determining the extent and prognosis of lesions, and thus for surgical treatment planning. Additionally, MRI allows for evaluating differential diagnoses, such as distinguishing osteomyelitis from NA.

In our experience, CT is less frequently used, typically limited to specific cases. It is useful in surgical treatment planning or post-treatment evaluation, especially when surgical hardware is in place.

## Types of injuries

### Neuropathic arthropathy

NA, also known as Charcot joint or Charcot neuroarthropathy, is a degenerative disease characterised by joint hypermobility and instability, bone and cartilage resorption, and extensive joint destruction [21]. It was first described in 1868 as a complication of *tabe dorsalis* by Jean Martin Charcot, who thought it resulted from damage to central-nervous-system trophic centres that controlled bone and joint nutrition [24]. Today, two main models of NA pathophysiology are proposed: the neurovascular and neurotraumatic theories.

According to the neurovascular theory, autonomic neuropathy causes increased blood flow by altering sympathetic tone and leads to increased bone resorption and osteopenia, allowing minor trauma on weakened subchondral bone to cause fractures, and joint collapse [21]. Moreover, the inflammation resulting from the fracture exacerbates hyperaemia, perpetuating the vicious cycle of increased bone turnover and joint destruction.

The neurotraumatic theory states that in the absence of normal protective sensory feedback, repetitive mechanical trauma causes progressive joint destruction [25]. The neurotraumatic model was supported by experiments on animals performed in the early 1900s in which severing the posterior nerve roots resulted in anaesthesia in the limbs of cats. Although anaesthesia itself did not result in arthropathy, when the cats were subjected to repeated trauma, they showed typical neuropathic joint lesions [26].

The real explanation is probably a combination of the two models: the lack of sensitivity allows for an extreme range of motion, thereby exposing a joint already weakened by increased bone turnover (due to autonomic neuropathy) to repeated microtrauma.

NA can virtually involve any joint. In a population of 14 children and young adults with CIP, 87% of Charcot joints occurred in lower limbs, with the most affected sites being, in order of frequency, the ankle, knee and hip, spine and elbow (the latter two being rarer but still significant locations) [2]. NA of the ankle, knee, hip and elbow have overlapping clinical presentation and imaging findings, whereas arthropathy of the spine deserves separate consideration.

### Ankle, knee, hip and elbow

#### Clinical presentation

Early stages clinically exhibit soft-tissue warmth, erythema and swelling, tending to be large, persistent and recurrent. Although pain is usually absent, patients may complain of minor discomfort or even no functional impairment [16, 27].

#### Imaging findings

**Fig. 1:**
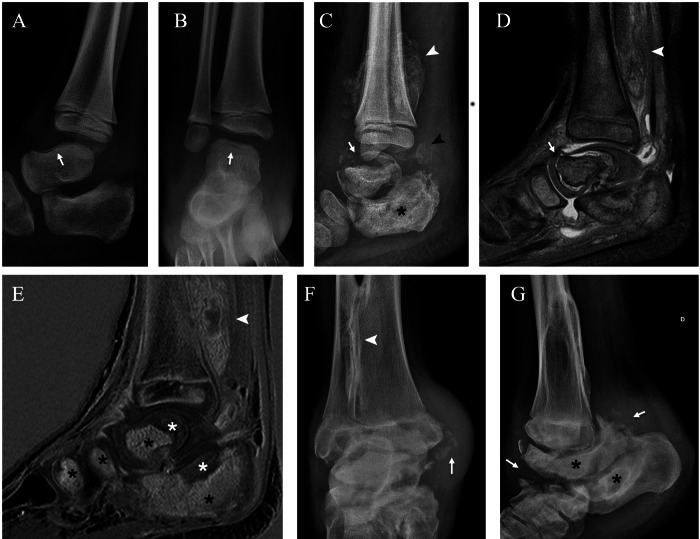
Neuropathic arthropathy of the right ankle in a boy with hereditary sensory and autonomic neuropathy type IV. Lateral (**A**) and anteroposterior (AP) (**B**) radiographs at 6 years of age show a linear cortical fracture of the talus (arrows). Lateral radiograph 1 month after the first set of radiographs (**C**) shows worsening of talar fractures (arrow), sclerotic and lytic changes of the calcaneus (asterisk) and loose bone fragments (black arrowhead); note the exuberant callus due to a fibular fracture (white arrowhead). Sagittal proton density (PD) (**D**) and T1 post-gadolinium MRI (**E**) show the talar fractures delineated by the fluid-filled cleft on the articular surface (arrow) and a diffuse enhancement of tarsal bones (black asterisk), respectively, in contrast to non-enhanced subchondral areas of sclerosis (white asterisk). The fibular callus is evident behind the tibia with increased enhancement (white arrowhead). AP (**F**) and lateral (**G**) radiographs at 14 years of age show permanent deformity with collapse of the tibio-talar and talo-calcaneal joints, talar and calcaneal sclerosis and flattening (asterisk), loose bone fragments (arrow) and tibio-fibular fusion (arrowhead)

**Fig. 2:**
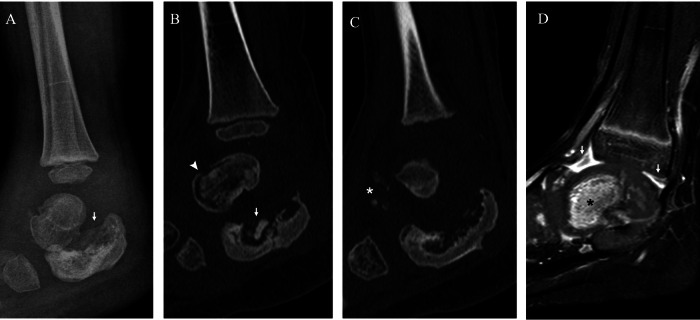
Neuropathic arthropathy of the right ankle in a 3-year-old boy with hereditary sensory and autonomic neuropathy type IV. Lateral radiograph at injury (**A**) shows sclerotic and lytic changes of the calcaneus with bone collapse (arrow). Sagittal CT (**B**, **C**) confirms fragmentation of the calcaneus (arrow) and shows subcortical fracture of the talus (arrowhead) and intra-articular loose bodies (white asterisk). Sagittal T1-weighted post-gadolinium MRI (**D**) shows bone marrow enhancement of the talus (black asterisk) and diffuse synovial hyperenhancement (arrows)

**Fig. 3:**
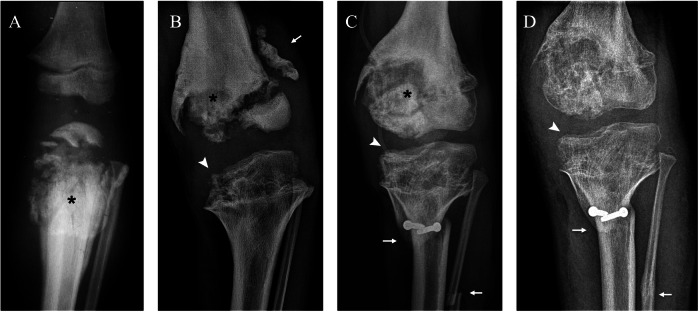
Neuropathic arthropathy of the left knee of a boy with hereditary sensory and autonomic neuropathy type IV. Anteroposterior radiographs at 3 (**A**), 5 (**B**) and 7 (**C**, **D**) years of age. Radiograph at injury (**A**) shows fragmentation of the tibial proximal metaphysis (asterisk). Radiograph (**B**) shows collapse of the tibial medial plate (arrowhead), lytic changes and fragmentation of the distal femoral epiphysis and metaphysis (asterisk) and bone fragments (arrow). Radiograph after oblique proximal tibial osteotomy (arrows) (**C**) shows partial healing of the tibial plate (arrowhead) and sclerosis of the femoral epiphysis (asterisk). Radiograph 2 months after surgery (**D**) shows progressive healing of the osteotomies (arrows) and the tibial plate (arrowhead)

**Fig. 4:**
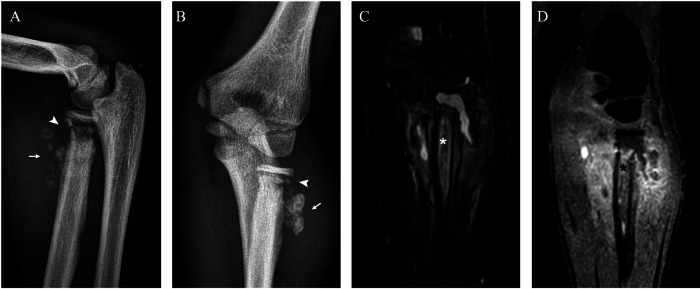
Neuropathic arthropathy of the left elbow of a 9-year-old boy with hereditary sensory and autonomic neuropathy type IV. Lateral (**A**) and anteroposterior (**B**) radiographs show lysis of the radial proximal metaphysis (arrowhead) and bone fragments (arrow). Coronal proton density (**C**) and post-gadolinium MRI (**D**) show oedema (white asterisk) and enhancement (black asterisk) of the proximal radius and surrounding soft tissues

**Fig. 5:**
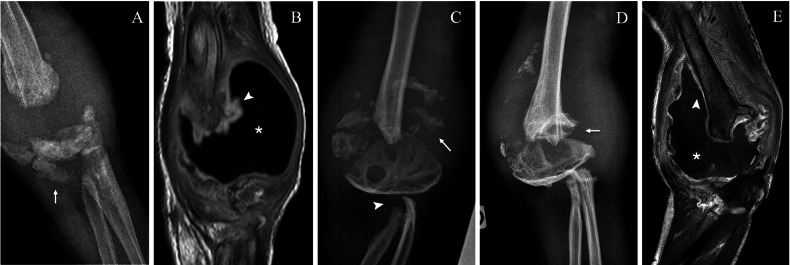
Neuropathic arthropathy of the right elbow of the same boy as in Fig. 4 with bilateral involvement of the elbows. Anteroposterior (AP) radiograph (**A**) and coronal T1-weighted post-gadolinium MRI (**B**) at 2 years of age during an episode of septic arthritis complicating the pre-existing NA, showing fragmentation of the distal humeral epiphysis (arrow), synovial thickening and enhancement (arrowhead) and joint effusion (asterisk). AP radiograph at age 6 years (**C**) shows partial healing of the humeral epiphysis with persistent fragmentation (arrow) and resorption of the proximal radius and ulna (arrowhead). AP radiograph (**D**) and coronal T1-weighted post-gadolinium MRI (**E**) show distal humeral pseudoarthrosis (arrow), joint affusion (asterisk) and synovial thickening and enhancement (arrowhead) with a sterile joint fluid aspiration

Plain radiographs can reveal subchondral fractures and joint effusion at early stages (Figs. 1–4) (with the exception of hip joint effusions, which are difficult to recognise on plain radiographs and more easily identified on ultrasonography). Late stages exhibit metaphyseal and epiphyseal partial collapse with fragmentation and sclerosis (Figs. 1–3, 5). In the acute phase, MRI shows bone marrow oedema and enhancement, cartilaginous damage, joint effusion and soft-tissue oedema [11] (Fig. 4). In the chronic stage, bone marrow demonstrates a low signal intensity in the subchondral bone on both T1- and T2-weighted images and no enhancement, a finding radiographically associated with osteosclerosis (Figs. 1, 2). CT allows for accurate detection of intra-articular loose bodies to plan surgical debridement (Fig. 2C).

#### Differential diagnosis

In the acute phase, NA subchondral fracture and common fracture are identical, except for their clinical presentation. Differentiating NA and osteomyelitis is often difficult, and both may coexist in the same joint. On MRI, both entities may demonstrate bone marrow oedema and enhancement, joint effusion and surrounding soft-tissue oedema. However, since NA is primarily an articular disease, the oedema tends to be juxta-articular, centred on subchondral bone and involving several bones, whereas the oedema in osteomyelitis is confined to a unique site, tending to be more diffuse and on one side of the joint [28]. Secondary signs of osteomyelitis, such as skin ulceration, cellulitis, soft-tissue abscess or sinus tracts, can improve the diagnostic accuracy.

Synovial thickening and enhancement may point to septic arthritis but can also be observed in sterile NA. Only joint aspiration or bone biopsy can give a definite answer (Fig. 5).

Two other possible differential diagnoses in children are haemophilic arthropathy and juvenile idiopathic arthritis, which both lead to bone and cartilage destruction at advanced stages. Diagnosis is based on clinical history because patients lack sensory deficit. In addition, clinical history of severe factor VIII or IX deficiency and the presence of hemosiderin deposits on MRI easily orient the diagnosis in the case of haemophilic arthropathy [29].

Another differential diagnosis specific to hip NA is Legg-Calvé-Perthes disease, with changes primarily focused on the femur, characterised by progressive widening and flattening of the femoral head, followed by fragmentation and destruction, in contrast to NA, where the process involves the entire joint and results in significant destruction of the acetabulum.

#### Treatment

The aim of treatment in patients with NA is to relieve pressure on the joints, preserve normal joint axis, and prevent progression to deformities. Orthosis and reduced activity are used to protect the joints. However, poor healing, non-union, pseudarthrosis and progressive deformity are common [9]. Surgery may be indicated in selected cases, and possible options include arthrotomy for debridement of loose bodies and necrotic fragments, arthrodesis and correction of severe deformities [16–18]. Arthroplasty is avoided because of the high rate of complication [5].

### Spine

#### Clinical presentation

Symptoms and signs of Charcot spine in patients with sensory deficit include numbness and muscle weakness in the lower extremities, walking inability and progressive spine deformity. Examination reveals lower-limb hyposthenia and decreased deep tendon reflexes [30, 31].

#### Imaging findings

**Fig. 6:**
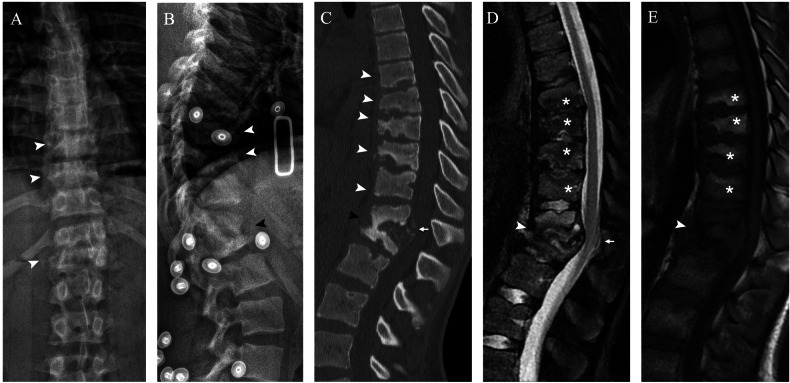
Neuropathic arthropathy of the thoracolumbar spine in a 15-year-old boy with hereditary sensory and autonomic neuropathy type IV, who presented intermittent claudication. Anteroposterior (**A**) and lateral (**B**) radiographs of the dorso-lumbar spine showing lytic changes of multiple vertebrae (white arrowhead) and collapse of T12-L1 (black arrowhead). Sagittal CT (**C**) confirms endplate destruction from T7 to T11 (white arrowhead), L1 collapse with partial fusion of T12-L1 (black arrowhead), and intracanal bone fragments (arrow). Sagittal T2 (**D**) and T1 (**E**) MRI confirm the intracanal protrusion of the posterior wall of L1 (arrow), fragmentation and oedema of T12-L1 (white arrowhead) as well as endplate lytic changes and degenerative fatty changes of bone marrow in multiple thoracic vertebrae (asterisk)

The thoracolumbar junction and lumbar spine are most frequently affected, with one or more segments being involved. Radiographs show lytic and sclerotic changes of the vertebrae and disc space narrowing (Fig. 6A, B). CT demonstrates sclerosis, lysis, fragmentation and height loss of vertebral bodies; displacement of fragments into paraspinal musculature and spinal canal; and paraspinal soft-tissue calcifications (Fig. 6C). MRI is essential to assess intracanal protrusion of the disc and bone fragments, radicular conflict, and spinal cord compression (Fig. 6D, E). All imaging modalities are used for diagnosis and for post-surgical follow-up and complications.

#### Differential diagnosis

The involvement of all three vertebral columns with destruction of the facet joints and the multilevel extension are signs that help differentiate spine NA from osteomyelitis, in which only one column is usually involved, and sclerosis and fragmentation are limited to the adjacent end plates [25]. Pott disease is an exception, because it presents a more extensive destruction; abscesses of the surrounding soft tissues are usually associated and help make the diagnosis. Two other differential diagnoses of osteolytic lesions and vertebral collapses in children are acute leukaemia and chronic recurrent multifocal osteomyelitis (CRMO). In acute leukaemia, diffuse bone demineralisation is frequently associated. In CRMO, other lesions in the metaphysis of long bones, particularly the tibia, may be present.

#### Treatment

In most cases in the literature, surgical fusion is performed to correct deformity and prevent further neurological sequelae. Progressive radiographic destruction and the need for revision surgery have been described [31].

### Joint dislocation

Joint subluxation and dislocation may occur at late stages of NA, as a consequence of increased ligamentous laxity and destruction of osteochondral surfaces [16] or without pre-existing arthropathy on intact joints. The hip is the most common location [2, 13]. Video gait analysis of young CIP patients indicated that they walk fast, with a long stride, but without smooth heel deceleration before foot contact with the floor, which has been speculated to contribute to the high incidence of joint dislocations and fractures in the lower limbs [32].

#### Clinical presentation

Joints are painless and mobile, so the only presenting symptom may be a limp noticed by the parents or an abnormal range of motion at physical examination.

#### Imaging findings

**Fig. 7:**
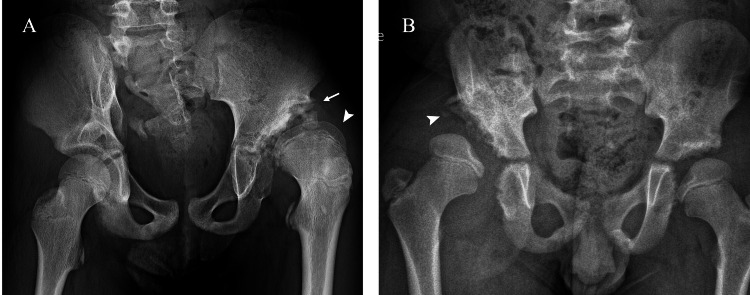
Neuropathic arthropathy (NA) of the left hip in a boy under treatment for lipomyelomeningocele at birth (**A**). Anteroposterior (AP) pelvic radiograph at 10 years of age (**A**) shows flattening of the femoral head and thickening of the femoral neck (arrowhead), superior subluxation and the formation of a neo-acetabulum (arrow) due to chronic dislocation. Dislocation of the right hip without underlying NA in an 8-year-old boy with hereditary sensory and autonomic neuropathy type IV (**B**). AP radiograph (**B**) shows lytic changes of the iliac bone (arrowhead) without anomalies of the femoral head

In hip dislocation, radiographs show the displacement of the femoral head relative to the acetabulum (Fig. 7B). No subchondral bone lesions typical of NA are observed. With chronic dislocation, a neo-acetabulum formation may be found, indicating the development of a secondary acetabular structure. MRI allows for a detailed evaluation of soft-tissue structures and joint effusion and assessment of the extent of the neo-acetabulum.

#### Differential diagnosis

Congenital hip dysplasia should not be difficult to differentiate given its distinct age of presentation and the absence of associated neuropathy.

#### Treatment

Patients with no underlying arthropathy tolerate hip dislocation for decades with minimal limitations and no consequences on their ambulatory status [33]. Additionally, reconstructive hip surgeries have a high rate of failure, so hip subluxations and dislocations should be treated with watchful waiting [13].

### Avascular necrosis

AVN may occur in both proximal and distal femoral epiphyses and is more frequent in the distal epiphysis. AVN is the most commonly reported cause of knee deformity in children with HSAN [13]. Paediatric AVN of the lateral femoral condyle was first described in patients with sensory deficit resulting from SDs and surgery for spinal neuroblastoma [20, 34]. Joint involvement may be unilateral or bilateral [35]. Both lateral and medial femoral condyles can be involved, resulting in genu valgum or varum deformity, respectively [13].

#### Clinical presentation

Patients present conspicuous warmth and swelling, painless knee effusion, knee deformity, and difficulty walking.

#### Imaging findings

**Fig. 8:**
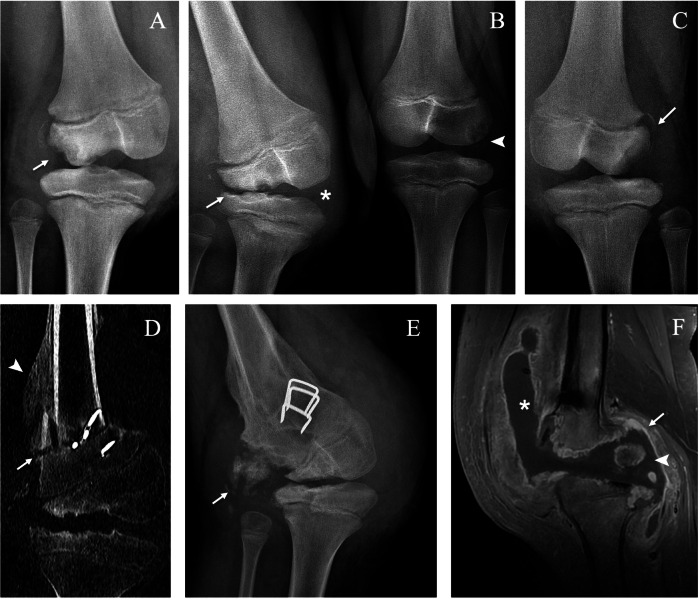
Bilateral knee avascular necrosis (AVN) in a boy with hereditary sensory and autonomic neuropathy type IV. Anteroposterior (AP) radiograph of the right knee at 9 years of age (**A**) shows bone resorption of the lateral femoral condyle (arrow). AP radiograph of both knees 3 months later (**B**) shows progression of the right lateral condyle resorption (arrow) with valgus deformation and widening of the medial articular space (asterisk) and lytic changes of the left lateral condyle (arrowhead). AP radiograph of the left knee after 3 more months (**C**) demonstrates progression of the osteolysis (arrow). Coronal CT of the right knee (**D**) after distal femoral varus osteotomy (arrow) shows signs of consolidation (arrowhead). AP radiograph of the right knee 1 year after surgery (**E**) shows recurrence of AVN of the lateral condyle (arrow). Sagittal T1 post-gadolinium MRI (**F**) during an episode of septic arthritis shows nonspecific findings such as joint effusion (asterisk), synovial enhancement (arrow) and intra-articular loose bodies (arrowhead)

**Fig. 9:**
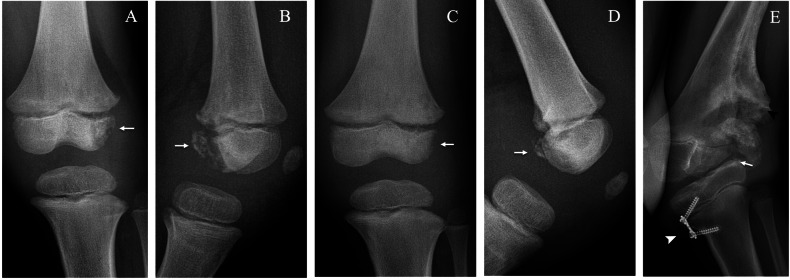
Left knee avascular necrosis in the brother of the patient shown in Fig. 8 at 5 years of age. Anteroposterior (AP) (**A**) and lateral (**B**) radiographs at injury show fragmentation of the lateral femoral condyle (arrow). AP (**C**) and lateral (**D**) radiographs at 21 days show partial healing of the lesion (arrow). AP radiograph at 9 years of age (**E**) shows progression of the bone resorption in the lateral condyle (arrow) with abundant callus formation (black arrowhead) after medial tibial hemi-epiphysiodesis (white arrowhead)

Plain radiographs demonstrate bone fragmentation of the femoral condyle (lateral or medial) along with increased joint space of the opposite compartment (medial or lateral) and knee effusion (Figs. 8, 9). Bone fragments may be seen scattered throughout the joint, consistent with intra-articular bodies. On MRI, the osteochondral fragment may be delineated by a fluid-filled cleft on the articular surface (rim sign); the presence of joint effusion and intra-articular bodies is also better assessed on MRI. Moreover, MRI allows for a differential diagnosis with septic arthritis, which is a possible complication of chronic AVN (Fig. 8F).

#### Differential diagnosis

Patients with spontaneous AVN of the knee without sensory deficit report pain on the inside of the knee, and as the disease progresses, they can no longer stand and bear weight on the affected knee. However, CIP patients complain of minor discomfort or have no functional impairment.

#### Treatment

In early stages, protected weight bearing is obtained with orthosis and crutches. Knee deformities resulting from AVN are progressive and can preclude walking without aid if not corrected. Corrective osteotomies and hemi-epiphysiodesis have shown good results but with possible recurrence. The correction rate is lower as compared with patients with idiopathic genu valgum. In neuropathic patients, epiphysiodesis and shoe raising are preferred to lengthening procedures to treat leg-length discrepancy [13, 33]. Total arthroplasty is usually avoided because of the high predisposition to bone infections. In wheelchair-dependent patients, amputation should be considered in case of failure of the reconstructive procedures, as severe deformities can complicate care [12].

### Fractures

Multiple fractures are the most frequent orthopaedic complications in HSAN. They tend to heal with hyperplastic bone formation [12] and are mostly located in the lower extremities [13]. The most widely accepted explanation is that since patients do not experience pain, there are considerable delays in diagnosis and care, which leads to continuous motion and weight bearing at the site of fracture and prevents proper healing [5].

#### Clinical presentation

Fractures typically occur spontaneously or after a minor trauma. The only signs may be swelling and redness in the soft tissues around the fracture site [1].

#### Imaging findings

**Fig. 10:**
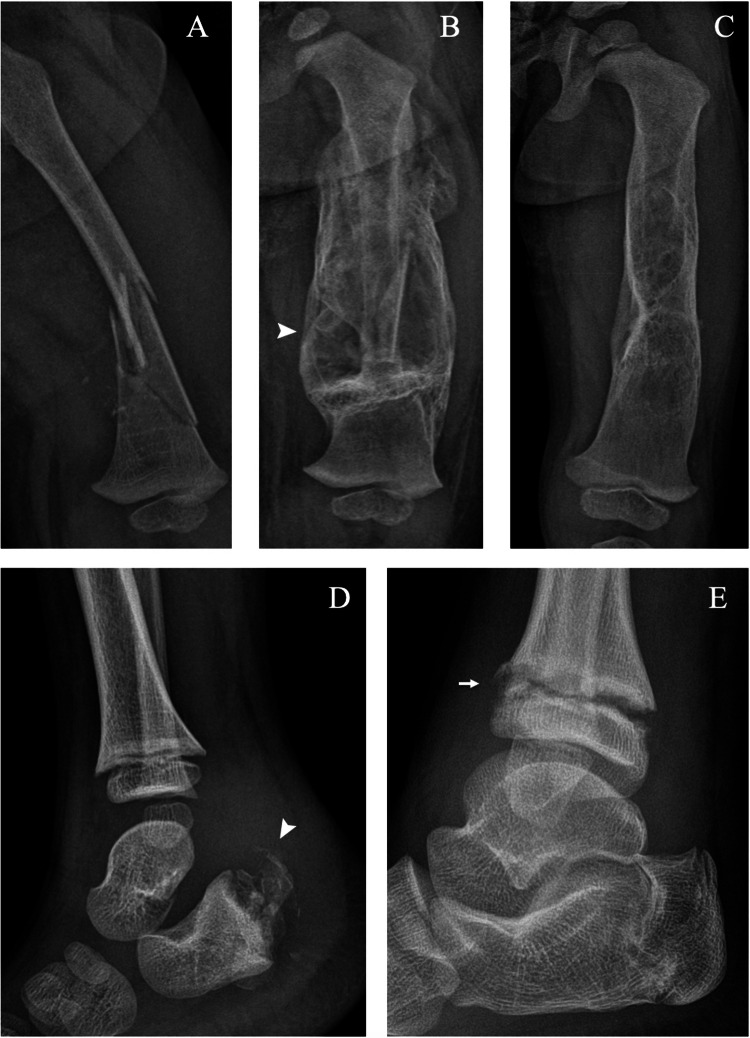
Two cases of lower-limb fractures of the left femur of a 1-year-old boy with congenital insensitivity to pain (**A**–**C**) and the right calcaneus of a 5-year-old boy with hereditary sensory and autonomic neuropathy type IV (**D**, **E**). Anteroposterior radiographs of the femoral fracture at injury (**A**), 3 months (**B**) and 13 months (**C**) show a large subperiosteal haematoma (arrowhead) (**B**) and the ongoing remodelling of the femoral diaphysis after complete healing (**C**). Lateral radiographs of the calcaneal fracture (arrowhead) at injury (**D**) and 10 years later (**E**) show complete healing without deformity; note the epiphyseal separation (arrow) of the tibia

Radiographs show fractures through the diaphysis or metaphysis of long bones healing with transverse orientation, and exuberant and bizarre callus formation [25] (Fig. 10A, B). Isolated calcaneus and talus fractures are also an omnipresent finding in most patients with CIP [5] (Fig. 10D, E).

#### Differential diagnosis

Non-accidental injury should always be considered in young children with multiple fractures and exuberant callus formation, although a normal sensory examination can help the diagnosis.

#### Treatment

Fractures were mostly managed non-operatively in all case series. Immobilisation is accomplished with casting. Surgical fixation is rarely needed.

### Epiphyseal separation

Epiphyseal separation, also classified as Salter-Harris type I fracture, is a particular type of fracture that is common in patients with myelomeningocele and CIP [1, 11, 12].

#### Clinical presentation

Swelling and warmth around the joint without a history of trauma are the only signs [1].

#### Imaging findings

**Fig. 11:**
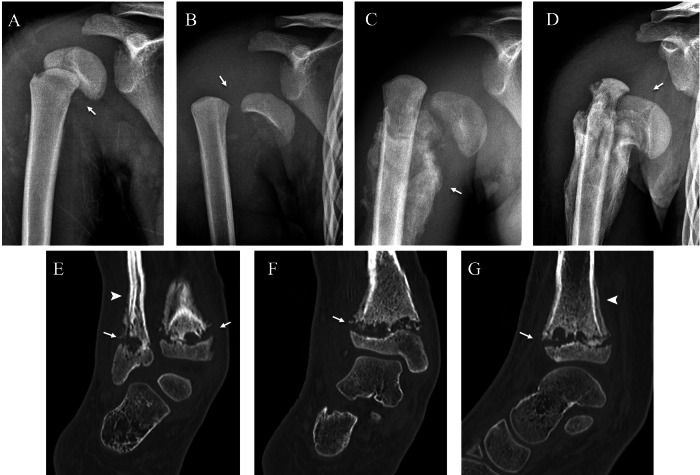
Two cases of epiphyseal separation in the right proximal humerus of a 5-year-old boy with hereditary sensory and autonomic neuropathy type IV (**A**–**D**) and the right distal tibia and fibula of a 9-year-old girl undergoing treatment for myelomeningocele (**E**–**G**). Anteroposterior right shoulder radiographs at injury (**A**), 1 week (**B**), 1 month (**C**), and 2 months (**D**) demonstrate, respectively, epiphyseal displacement (**A**), dislocation (**B**), excessive callus formation (**C**) and healing with resulting arm shortening (**D**) (arrows). Coronal (**E**, **F**) and sagittal (**G**) CT of the right ankle shows widening of the growth plate, irregularity and lytic areas of the metaphysis and epiphysis of the tibia and fibula (arrow), and periosteal reaction (arrowhead). Courtesy of Dr. Maher Yatim, Auxerre

Plain radiographs can show different ranges of epiphyseal displacement that heal with excessive callus formation (Fig. 11A–D). CT allows for a more detailed evaluation, showing in addition to the widening of the growth plate, irregularity and lytic areas of the metaphysis and epiphysis and periosteal reaction (Fig. 11F, G).

#### Treatment

Management is the same as for other types of fractures.

### Acro-osteolysis

Progressive resorption of the distal phalanges of hands and toes, also known as acro-osteolysis or auto-amputation, is a noteworthy feature in most cases of HSAN and usually begins in the first year of life [14]. The pathophysiology is not fully understood: in CIP patients, bone resorption is probably the result of repeated self-mutilation, biting and other microtrauma complicated by infection in insensate hands [25]. In SD patients, acro-osteolysis is exclusively seen in toes, along with other trophic changes in skin and nails secondary to sympathetic denervation [36].

#### Clinical presentation

Physical examination can reveal partial mutilation of the fingertips, absence of fingernails or signs of self-biting.

#### Imaging findings

**Fig. 12:**
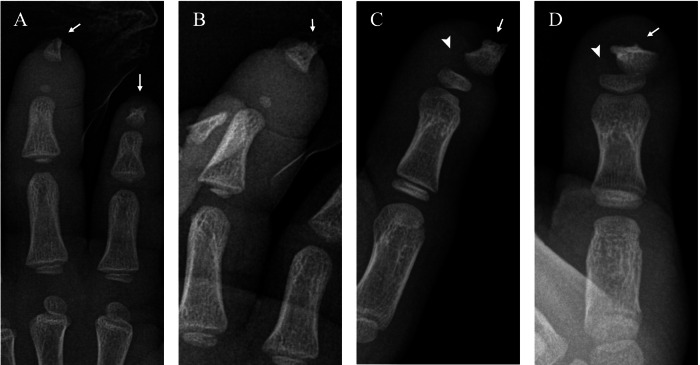
Acro-osteolysis in the left hand of a patient with hereditary sensory and autonomic neuropathy type IV at 2 (**A**, **B**) and 8 years of age (**C**, **D**). AP (**A**, **D**) and lateral (**B**, **C**) radiographs show bone resorption (arrows) of the distal phalanges of the 2nd and 3rd (**A**, **B**) and the 1st (**C**, **D**) fingers; note the epiphyseal separation (arrowhead) of the distal phalanx

Radiographs show lytic lesions of the distal phalanges with a transverse and relatively regular appearance of erosion, resulting in significant loss of substance (Fig. 12).

#### Differential diagnosis

The main differential diagnosis is infection, specifically osteitis, due to complication of paronychia; the diagnosis is clinical. Other causes of terminal phalangeal bone erosion in children are genetic disorders, particularly primary hypertrophic osteoarthropathy and skeletal dysplasia; rheumatic diseases, such as psoriatic arthritis and systemic sclerosis; and hyperparathyroidism [37]. Although psoriatic arthritis stands out as the cause of the primary destruction of the interphalangeal joint, which leads to resorption of the proximal end of the distal phalanx, all other aetiologies have a common radiological pattern of resorption of the terminal tuft. Therefore, diagnosis depends on normal sensory examination and other clinical features [37].

#### Treatment

Treatment is a conservative approach and treatment of the associated infection.

## Conclusion

Neuropathic skeletal injuries are common in children with sensory loss due to HSAN and are less frequent in those with SD. These injuries result from repeated microtrauma and painless damage, often without the child’s awareness: the injuries include NA, AVN, joint dislocations, fractures and acro-osteolysis.

Imaging findings of neuropathic injuries may be mistaken for conditions such as osteomyelitis, septic arthritis, or other disorders. However clinical history, anatomic location and specific imaging features can help differentiate these entities. Particularly in a child with little or no pain, subchondral fractures with joint effusion, advanced joint destruction and/or dislocation, repeated fractures with exuberant calluses, epiphyseal separation, and acro-osteolysis are all suggestive of neuropathic injuries.

Since neuropathic injuries are frequently asymptomatic or present with nonspecific symptoms, radiologists should recognise their imaging appearances in that they may provide the first clue to the underlying diagnosis.

Given the risk of deformity and severe disability, along with the limitations of both surgical and conservative treatments, early diagnosis and trauma prevention are crucial for this patient population.

## Abbreviations

AVN: Avascular necrosis
CIP: Congenital insensitivity to pain
CRMO: Chronic recurrent multifocal osteomyelitis
HSAN: Hereditary sensory and autonomic neuropathies
NA: Neuropathic arthropathy
SDs: Spinal dysraphisms
